# Anti-Hypertensive Property of an NO Nanoparticle in an Adenine-Induced Chronic Kidney Disease Young Rat Model

**DOI:** 10.3390/antiox12020513

**Published:** 2023-02-17

**Authors:** You-Lin Tain, Hung-Wei Yang, Chih-Yao Hou, Guo-Ping Chang-Chien, Sufan Lin, Chien-Ning Hsu

**Affiliations:** 1 Division of Pediatric Nephrology, Kaohsiung Chang Gung Memorial Hospital, Kaohsiung 833, Taiwan; 2 College of Medicine, Chang Gung University, Taoyuan 333, Taiwan; 3 Institute for Translational Research in Biomedicine, Kaohsiung Chang Gung Memorial Hospital, Kaohsiung 833, Taiwan; 4 Department of Biomedical Engineering, National Cheng Kung University, Tainan City 701, Taiwan; 5 Medical Device Innovation Center, National Cheng Kung University, Tainan City 701, Taiwan; 6 Department of Seafood Science, National Kaohsiung University of Science and Technology, Kaohsiung 811, Taiwan; 7 Center for Environmental Toxin and Emerging-Contaminant Research, Cheng Shiu University, Kaohsiung 833, Taiwan; 8 Institute of Environmental Toxin and Emerging-Contaminant, Cheng Shiu University, Kaohsiung 833, Taiwan; 9 Super Micro Mass Research and Technology Center, Cheng Shiu University, Kaohsiung 833, Taiwan; 10 Department of Pharmacy, Kaohsiung Chang Gung Memorial Hospital, Kaohsiung 833, Taiwan; 11 School of Pharmacy, Kaohsiung Medical University, Kaohsiung 807, Taiwan

**Keywords:** nitric oxide, chronic kidney disease, asymmetric dimethylarginine, hypertension, renin–angiotensin system, oxidative stress, nanoparticle

## Abstract

Hypertension is the most common complication of chronic kidney disease (CKD) in children but is still poorly controlled. Nitric oxide (NO) deficiency plays a pivotal role in CKD and hypertension. NO is known to have health benefits, while NO typically has a short half-life and is not specifically targeted. In this study, we used a pediatric CKD model, which was induced in young rats by feeding them 0.25% adenine. We investigated two different NO donors, namely S-nitrosoglutathione (GSNO) and diethylenetriamine/NO adduct (DETA NONOate) via intraperitoneal injection at 10 mg/kg/day daily for 3 weeks. GSNO was delivered by Cu^2+^-doped zeolitic imidazolate framework (Cu/ZIF-8) nanoparticles to generate NO. As a result, we observed Cu/ZIF-8 nanoparticles were successfully loaded with GSNO and were able to release NO. Young rats fed with adenine displayed kidney dysfunction and hypertension at 9 weeks of age, which were prevented by GSNO-loaded nanoparticle or DETA NONOate treatment. GSNO-loaded nanoparticles reduced CKD-induced hypertension, which was related to an enhanced endogenous NO-generating system, reduced renal oxidative stress, and downregulated several components belonging to the classic renin–angiotensin (RAS) system. Our results cast new light on targeting NO delivery through the use of nanoparticles aiming to improve child-focused outcomes related to CKD worthy of clinical translation.

## 1. Introduction

Hypertension is a well-recognized risk factor for cardiovascular disease and global death [1]. Accumulative evidence supports that hypertension takes its origins in early life [2,3]. In children, chronic kidney disease (CKD) is the leading cause of hypertension [4]. On the other hand, hypertension is the most prevalent complication in childhood CKD [5]. In pediatric CKD, prior work has indicated that even in early-stage CKD, more than 50% of children display elevated blood pressure (BP) load [6,7]. These observations suggest CKD and hypertension are intrinsically linked and both share pathological mechanisms. 

Many children with CKD have uncontrolled hypertension, even with multiple antihypertensive therapies [8]. Since CKD is a leading cause of resistant hypertension [9], the search after novel treatment for resistant hypertension is unremitting, especially for children with CKD. Nitric oxide (NO), a gasotransmitter, participates in the regulation of BP [10]. NO deficiency has been implicated in hypertension and CKD [11,12]. The causes of NO deficiency consist of substrate L-arginine deficiency, decreased abundance and/or activity of nitric oxide synthase (NOS), suppression by oxidative stress, and inhibition by asymmetric and symmetric dimethylarginine (ADMA and SDMA, both are endogenous NOS inhibitors), etc. [11,12]. Conversely, NO donors and NO-targeted therapies have shown beneficial effects in attenuating BP [13].

NO donors are pharmacologically active substances that have the ability to release NO [14]. S-nitrosoglutathione (GSNO) is an amino acid NO donor which, when under physiological conditions, spontaneously releases NO. Diethylenetriamine/NO adduct (DETA NONOate) is another NO donor, which has the longest NO generating half-life in vitro [15]. Although GSNO and DETA NONOate have shown vasodilatory effects [16,17], whether they can prevent CKD-induced hypertension remains basically unknown.

Recently, nanoparticle-based systems have been used for sustained NO delivery [18]. We, hence, developed Cu^2+^-doped zeolitic imidazolate framework (Cu/ZIF-8) nanoparticles to deliver GSNO for generating NO. Here, we aim to evaluate the anti-hypertensive effect of the GSNO-loaded Cu/ZIF-8 nanoparticles and DETA NONOate using an adenine-induced CKD young rat model. 

## 2. Materials and Methods

### 2.1. Synthesis of Cu-Doped ZIF-8

A solution of ZnCl_2_ and Cu(NO_3_)2 (5 mg/mL for each) in 10 mL of ethanol, 2-Methylimidazole (2-MIM; 240 mg) in 20 mL of ethanol, and poly(vinyl alcohol) (PVA; 1% *w/v*) in 1 mL of deionized water (DI-H_2_O) were separately equipped. These two solutions were then mixed in a three-neck flask by the dropwise addition of the Zn^2+^ and Cu^2+^ solution to the 2-MIM and PVA solution, which was conducted under nitrogen flow at room temperature with stirring for 6 h. The Cu-doped ZIF-8 nanoparticles (Cu/ZIF-8) were separated by centrifugation (12,000 rpm, 10 min) and washed with ethanol/DI-H2O (1:1 *v/v*; 3 × 10 mL). Samples were air-dried under ambient conditions overnight. To use the material in catalytic reactions, the Cu/ZIF-8 products were activated by treatment of the powders at 200 °C for 6 h and then naturally cooling to room temperature within the oven. The vials containing the Cu/ZIF-8 nanoparticles were tightly capped and stored at room temperature before use.

### 2.2. Preparation of GSNO-Loaded Cu/ZIF-8

To avoid the light-induced damage of GSNO, all the fabrication processes with GSNO were carefully performed in the dark. The GSNO loading into the Cu/ZIF-8 nanoparticles were performed by mixing different concentrations of GSNO with 1 mL of Cu/ZIF-8 nanoparticles (10 mg/mL) at room temperature for 1 h. The above mixture solution was then purified by centrifugation (12,000 rpm, 10 min) and washed three times with DI-H_2_O to obtain GSNO-loaded Cu/ZIF-8. We further measured the amount of unloaded GSNO in supernatant to determine the GSNO loading efficiency by absorbance of GSNO at 345 nm using a SpectraMax M2 microplate reader (Molecular Devices Co., San Jose, CA, USA) with the GSNO standard curve.

### 2.3. In Vitro NO Generation Measurement

In brief, 10 mg of GSNO-loaded Cu/ZIF-8 was put into a 15 mL centrifuge tube, with no GSNO-loaded Cu/ZIF-8 utilized as a control (*n* = 3). The final composition of the working solution was 100 μM glutathione (GSH). A working solution of 10 mL was added into each centrifuge tube and incubated for 1, 2, 3, 4, 5, and 6 h at 37 °C, under darkness and with gentle shaking. NO generation was determined at the indicated time points measured by using Griess reaction assay. We used 100 μM sodium nitrite as a standard and Di-H_2_O as a blank. Absorbance was measured at 550 nm using a microplate reader. 

### 2.4. Animal Model of CKD

Animal care and experiments were carried out following the Guide for the Care and Use of Laboratory Animals and were approved by the Institute of Animal Care and Use Committee at our hospital (Permit No. 2021081102; approval date: 15 February 2022). Animals were housed in an AAALAC-accredited animal center within the Kaohsiung Chang Gung Memorial Hospital. The protocol of CKD induction was based on our previous work [19]. Male Sprague–Dawley (SD) rats aged 3 weeks received regular chow (*n* = 8) or chow supplemented with 0.25 % adenine for 3 weeks (*n* = 32). 

**Figure 1:**
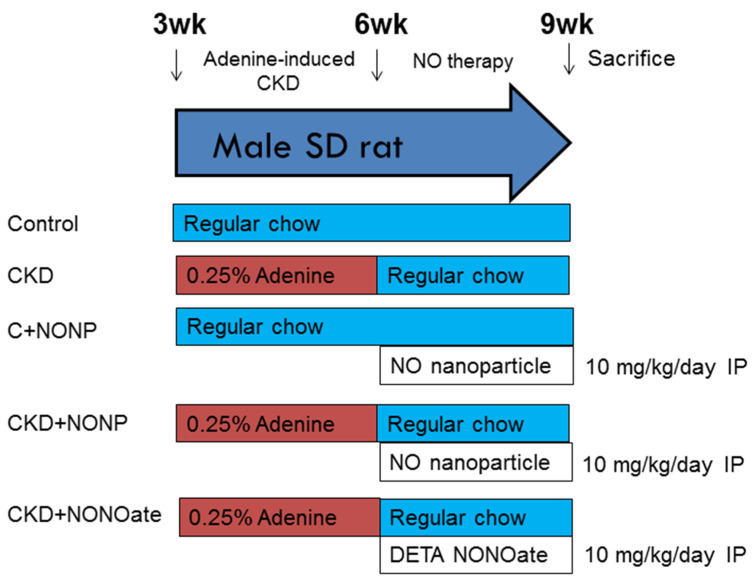
Experimental protocol used in the current study.

Figure 1 shows the experimental protocol. Rats were randomly assigned to five groups (*n* = 8/group): group 1: control, group 2: CKD, group 3: C+NONP, group 4: CKD+NONP, and group 5: CKD+NONOate. The rats in groups 3 and 4 received NO-releasing nanoparticle administration via intraperitoneal injection at 10 mg/kg/day for 3 weeks (week 4-6). Group 5 rats received DETA NONOate administration (10 mg/kg/dose; Merck Ltd., Taipei, Taiwan) via intraperitoneal injection daily for 3 weeks (week 4-6). The rationale and dosage of DETA NONOate utilized in the present study were based on the previous report [20]. 

Blood pressure (BP) was measured in conscious rats every two weeks using an indirect tail-cuff method (CODA, Kent Scientific Corp., Torrington, CT, USA). We trained the rats for a period of 1 week prior to starting the actual recording sessions by performing restraint and tail-cuff inflation. At nine weeks of age, rats were sacrificed. Heparinized blood samples were collected. We subsequently harvested the kidney samples and stored them at −80 °C in a freezer. Plasma creatinine concentrations were analyzed using high-performance liquid chromatography (HPLC, HP series 1100; Agilent Technologies Inc., Santa Clara, CA, USA).

### 2.5. Analysis of NO Parameters

We used a validated HPLC method for analysis of L-arginine and its methylated derivatives, as described previously [19]. These NO-related parameters were extracted from plasma by solid-phase extraction, derivatized with o-phthaldialdehyde containing 3-mercaptopropionic acid, and separated by reverse-phase chromatography using fluorescence detection. Homoarginine was used as the internal standard. The ratio of L-arginine to ADMA was calculated to represent an index of NO bioavailability [21]. 

### 2.6. Western Blot

We determined protein abundance of NO-generating enzyme endothelial NOS (eNOS) and neuronal NOS (nNOS), ADMA-metabolizing enzyme dimethylarginine dimethylaminohydrolase-1 and -2 (DDAH1 and DDAH2) by Western blot. Kidney cortical proteins in equal volumes (200 μg) were loaded on a polyacrylamide gel and separated by electrophoresis, followed by transferring onto nitrocellulose membranes. We next incubated the membranes with diluted primary antibodies (Table 1) and secondary antibodies. Immunoreactive bands were detected by enhanced chemiluminescence (PerkinElmer, Waltham, MA, USA) and quantified by Quantity One Analysis software (Bio-Rad, Hercules, CA, USA) as integrated optical density (IOD). The IOD was factored for Ponceau red staining to correct for any variations in total protein loading. The relative protein abundance compared with the control was calculated. 

### 2.7. Detection of Oxidative Stress by 8-OHdG Immunostaining

The occurrence of oxidative DNA damage was determined by immunostaining of 8-hydroxydeoxyguanosine (8-OHdG) [22]. The kidney sections were deparaffinized in xylene and subsequently rehydrated through graded alcohol. After blocking with immunoblock (BIOTnA Biotech., Kaohsiung, Taiwan), an anti-8-OHdG antibody (1:100, JaICA, Shizuoka, Japan) was added to the kidney sections for 2 h incubation, followed by the polymer–horseradish peroxidase (HRP)/3,3′-diaminobenzidine (DAB)-based detection. Positively 8-OHdG-stained cells were counted in five fields randomly selected from each kidney section using a light microscope (Nikon, Melville, NY, USA) at × 200 magnification.

### 2.8. Quantitative PCR

Total RNA was isolated from the rat kidney cortex tissue, as described earlier [18]. Two-step quantitative PCR was conducted by using the Quantitect SYBR Green PCR Reagents kit (Qiagen, Valencia, CA, USA) on the iCycler iQ Real-Time PCR Detection System (Bio-Rad, Hercules, CA, USA). Several RAS components were determined, including angiotensinogen (Agt), renin, (pro)renin receptor (PRR), angiotensin converting enzyme-1 (ACE1) and -2 (ACE2), angiotensin II type 1 receptor (AT1R), and angiotensin (1–7) MAS receptor (MAS). The 18S ribosomal RNA (R18S) gene was used as the internal control. Table 2 provides the PCR primer sequences. Samples were run in replicates. We performed the comparative threshold cycle (Ct) method for calculating relative gene expression values. The fold increase in the experimental sample, relative to the control, was calculated based on the formula 2^−ΔΔCt^. 

### 2.9. Statistical Analysis

All data are presented as the mean ± standard error of means (SEM). Analysis of difference was determined using one-way analysis of variance (ANOVA) and post-hoc Tukey test. Differences were considered significant at *p* < 0.05. 

## 3. Results

### 3.1. GSNO-Loaded Cu/ZIF-8 Nanparticle Characterization

**Figure 2:**
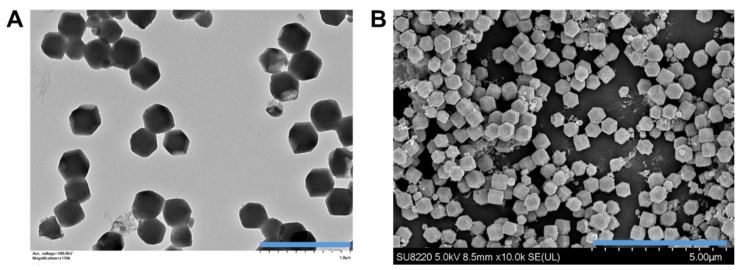
(**A**) Transmission electron microscopy (TEM) image (scale bar: 1 μm) and (**B**) scanning electron microscopy (SEM) image (scale bar: 5 μm) of GSNO-loaded Cu/ZIF-8 nanoparticles.

The GSNO-loaded Cu/ZIF-8 nanoparticles were synthesized and then were observed under transmission electron microscopy (TEM) and scanning electron microscopy (SEM). Figure 2 illustrates the GSNO-loaded Cu/ZIF-8 nanoparticles, which were rather small, and monodispersed nanoparticles with a well-defined truncated rhombic dodecahedron structure with the side length approximately 381 ± 17.6 nm. The successful loading of GSNO was also confirmed by the presence of an absorption peak originating from S–NO bonds at 345 nm. The loading amount of GSNO in the Cu/ZIF-8 nanoparticles was approximately 24.7 ± 3.2 wt% (0.247 mg GSNO/mg Cu/ZIF-8).

**Figure 3:**
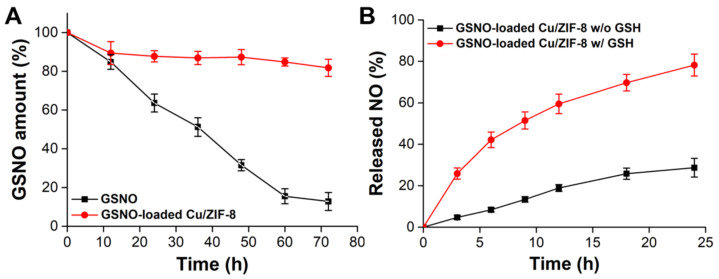
(**A**) Stability of GSNO and GSNO-loaded Cu/ZIF-8 in PBS solution (pH 7.4) without containing GSH. (**B**) NO release profiles from GSNO-loaded Cu/ZIF-8 in the absence and presence of 10 mM GSH. *n* = 3 experiments.

It is known that GSNO suffers from poor stability in aqueous solutions and tends to decompose to generate NO. Therefore, we entrapped the GSNO inside the Cu/ZIF-8 to stabilize the GSNO in the aqueous phase. Figure 3A illustrates the dissociation profiles of NO from GSNO and GSNO-loaded Cu/ZIF-8 in PBS solution without GSH (pH 7.4). More NO releasing from GSNO indicated the poor stability of GSNO. After 12 h, the dissociation of GSNO was not observed in the GSNO-loaded Cu/ZIF-8 group. The initial 10.6 ± 5.8% of dissociation may be due to GSNO adsorbed on the surface not inside the pores of the Cu/ZIF-8. In contrast, approximately 87.2 ± 4.6% of GSNO was dissicated after 72 h of incubation. These results suggested that the mesoporous Cu/ZIF-8 effectively shielded GSNO against water, and hence rationally enhanced the stability of GSNO. 

Our system revealed NO release from the GSNO-loaded Cu/ZIF-8 occurred in two steps: (i) dissolution of the ZIF-8 scaffold in acidic endosomes triggered GSNO and Cu^2+^ release and (ii) the released Cu^2+^ catalyzed the released GSNO to accerate NO generation in the presence of GSH. The release of NO from the GSNO-loaded Cu/ZIF-8 in the absence or presence of GSH was determined. As shown in Figure 3B, in the absence of GSH, release of NO was delayed to a level of 28.7 ± 4.5% after 24 h of incubation. In contrast, the Cu^2+^ promotes the decomposition of GSNO to generate more NO generation in the presence of GSH; up to 78.2 ± 5.3% of NO was released within 24 h, most likely because the GSH could reduce the Cu^2+^ to Cu^+^ which is the active agent to accerate GSNO decomposition for NO generation [23]. The results demonstrate that the GSNO-loaded Cu/ZIF-8 can not only effectively enhance the stability of GSNO in aqueous solution, but also promote the conversion efficiency of GSNO into NO and also generate NO from endogenous S-nitrosothiols.

### 3.2. Effects of NO Nanoparticles and DETA NONOate on Renal Outcomes

**Figure 4:**
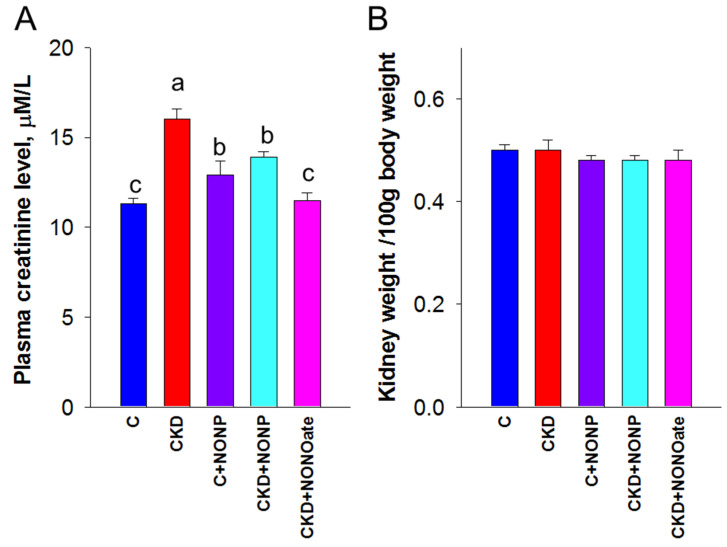

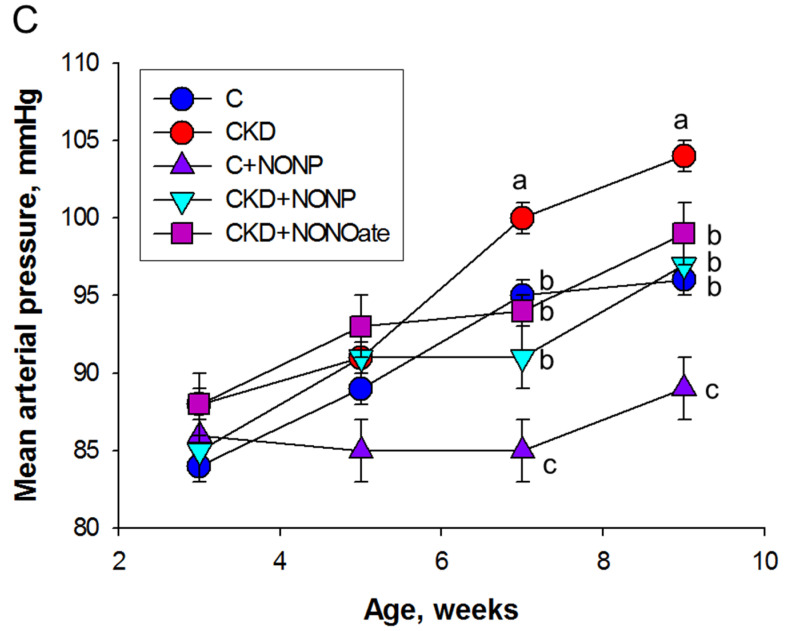
NO nanoparticle treatment attenuated kidney dysfunction and reduced blood pressure. (**A**) Plasma creatinine level. (**B**) The ratio of kidney weight to body weight was calculated to reveal the profile of renal hypertrophy. (**C**) Mean arterial pressure in rats from 3 to 9 weeks of age. MA. The letters a, b, and c indicate the differences between the groups (*p* < 0.05, one-way ANOVA); *n* = 8/group.

We first examined the effects of NO nanoparticles and DETA NONOate on renal outcomes. Renal function was determined by plasma creatinine (Cr) level (Figure 4A). Adenine-fed rats experienced a ~40% loss in kidney function (Cr: CKD vs. C = 16 ± 0.6 vs. 11.3 ± 0.3 μM; *p* < 0.05). The plasma concentration of Cr was reduced by NO nanoparticle treatment in the CKD+NONP group yet remained higher than that in the CKD+NONOate and C group. Renal hypertrophy was evaluated by the ratio of kidney weight to body weight (Figure 4B). In comparison, this ratio exhibited no differences among the five groups. Figure 4C shows that mean arterial pressure was increased in adenine-fed rats aged 9 weeks that became significant at seven weeks of age. The elevation of BP was similarly reduced in the CKD+NONP and CKD+NONOate group. At 9 weeks of age, NONOate treatment caused a reduction in BP in the CKD+NONOate group compared with the controls. In all, our data indicate that NO nanoparticles and NONOate have a similar BP-lowering effect, while NO nanoparticles protect against CKD progression, inferior to NONOate. 

### 3.3. Effects of NO Nanoparticles and DETA NONOate on NO Pathway

**Figure 5:**
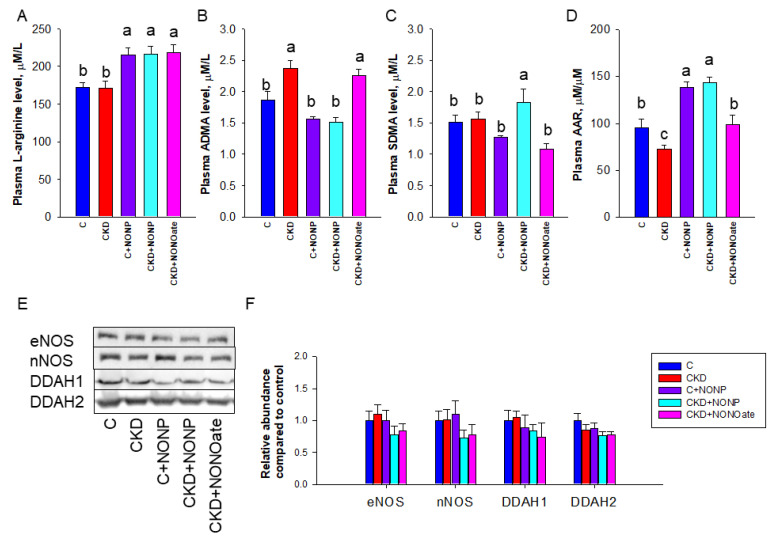
NO nanoparticle and DETA NONOate treatment resulted in altered NO pathway. Effects of NO nanoparticle treatment on plasma concentrations of (**A**) L-arginine, (**B**) asymmetric dimethylarginine (ADMA), (**C**) symmetric dimethylarginine (SDMA), and (**D**) the ratio of L-arginine to ADMA (AAR). (**E**) Representative Western blots demonstrate endotheial nitric oxide synthase (eNOS, 140 kDa), neuronal nitric oxide synthase (nNOS, 155 kDa), dimethylarginine dimethylaminohydrolase-1 (DDAH1, 31 kDa), and dimethylarginine dimethylaminohydrolase-2 (DDAH2, 30 kDa) bands. (**F**) The relative protein abundance of renal cortical eNOS, nNOS, DDAH1, and DDAH2 were calculated. The letters a, b, and c indicate the differences between the groups (*p* < 0.05, one-way ANOVA); *n* = 8/group.

As NO is generated by NOS isoenzymes and NOS can be inhibited by ADMA, we next examined these NO-related parameters (Figure 5). Compared with control and CKD rats, NO nanoparticles and DETA NONOate similarly increased plasma L-arginine concentration (Figure 5A). CKD led to increased plasma ADMA concentration, which was prevented by NO nanoparticle treatment (Figure 5B). Compared with other groups, SDMA level was higher in the CKD+NONP group (Figure 5C). Regarding AAR, the C+NONP and CKD+NONOate group had higher ratios than those in the C and CKD+NONOate group, while the CKD group had the lowest ratios (Figure 5D). We further examined NOS isoenzyme expression in the kidneys and found renal expressions of eNOS and nNOS were unchanged in response to NO nanoparticle or DETA NONOate treatment (Figure 5E,F). Similarly, the abundance of ADMA-metabolizing enzyme DDAH1 and DDAH2 in the kidneys was not altered by both NO doners (Figure 5E,F).

### 3.4. Effects of NO Nanoparticles and DETA NONOate on Oxidative Stress

**Figure 6:**
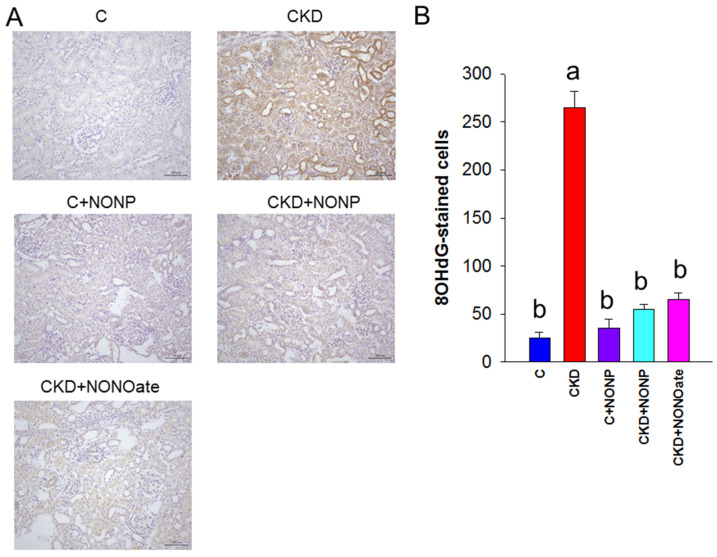
(**A**) Representative micrographs of kidney sections demonstrated significant 8-OHdG-positive cells in glomeruli and tubular cells in CKD group as compared with the other groups. (**B**) Quantitative analysis of 8-OHdG-positive cells per microscopic field (200×). The letters a and b indicate the differences between the groups (*p* < 0.05, one-way ANOVA); *n* = 8/group.

As NO has been shown to reduce oxidative stress and prevent CKD progression, we sought to examine whether NO nanoparticle treatment can reduce CKD-induced oxidative stress damage by evaluating oxidative DNA damage marker 8-OHdG [22]. As shown in Figure 6, there was intense kidney glomerular and tubular staining of 8-OHdG in the CKD group, as compared with weak staining in the other four groups. 

### 3.5. Effects of NO Nanoparticles and DETA NONOate on the RAS

**Figure 7:**
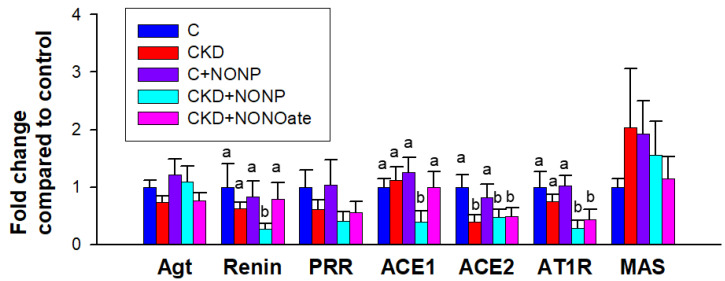
NO nanoparticle treatment caused alterations in the renin–angiotensin system (RAS). Barplots represent renal mRNA expression of RAS components, including angiotensinogen (Agt), renin, (pro)renin receptor (PRR), angiotensin converting enzyme-1 (ACE1) and -2 (ACE2), angiotensin II type 1 receptor (AT1R), and angiotensin (1–7) MAS receptor (MAS). The letters a and b indicate the differences between the groups (*p* < 0.05, one-way ANOVA); *n* = 8/group.

We further assessed the renal expression of RAS components as the interplay between RAS and NO is involved in hypertension; NO antagonizes the vasoconstrictive effect of angiotensin II (Ang II), whereas Ang II reduces NO bioavailability by promoting oxidative stress [24]. As shown in Figure 7, CKD diminished renal expression of ACE in the CKD, CKD+NONP, and CKD+NONOate group. NO nanoparticle treatment caused a decrease in renin, ACE1, and AT1R expression in the kidneys. DETA NONOate decreased renal expression of AT1R. The expression of Agt, PRR, and MAS in the kidney did not differ among the five groups (Figure 7).

## 4. Discussion

The results of the present study, with a young rat model of CKD, demonstrate that the NO donor GSNO-loaded nanoparticles and DETA NONOate counteracted the detrimental effects of CKD not only on BP but kidney function as well. The most significant findings of the present study can be summarized as follows: (1) Cu/ZIF-8 nanoparticles were successfully loaded with NO donor GSNO and were able to control the release of NO; (2) CKD rats developed hypertension at 9 weeks of age and this was prevented by GSNO-loaded nanoparticle or DETA NONOate therapy; (3) GSNO-loaded nanoparticles restored CKD-induced increased plasma ADMA level, decreased AAR, and renal oxidative damage; (4) GSNO-loaded nanoparticles reduced renal mRNA expression of renin, ACE1, and AT1R; and (5) the beneficial effect of DETA NONOate is related to increased plasma AAR, decreased renal oxidative damage, and downregulated renal AT1R expression.

Systemic NO bioavailability was decreased in adenine-treated CKD rats, characterized by increased ADMA concentration and decreased AAR in the plasma. Nowadays, several NO-related therapies have been reported for the treatment and prevention of hypertension and/or kidney disease, such as supplementation of NO precursor arginine or citrulline, ADMA-lowering agents, NO donors, augmentation of activity of NOS, etc. [25]. Although arginine supplementation is commonly used to generate NO in experimental studies [26], arginine is not a good NO precursor because of its multiple metabolic fates [27]. Citrulline is the precursor of arginine whilst avoiding its hepatic metabolism [28]. Accordingly, citrulline has promised as an effective therapy in many diseases related to NO deficiency. As the conversion of citrulline into arginine mainly happens in the kidney, this approach may not be an optimal way to treat CKD-induced hypertension. Additionally, a number of currently used drugs are able to lower ADMA levels and enhance NO bioavailability in experimental hypertension studies [29]. However, a specific ADMA-lowering agent is still inaccessible in clinical practice. We, hence, determined the anti-hypertensive effect of NO donors on CKD-induced hypertension in the current study.

Consistent with the properties of NO donors reported previously [14], we found CKD-induced hypertension and kidney dysfunction accompanied by the reduction of NO bioavailability were restored by either GSNO-loaded nanoparticle or DETA NONOate treatment. Although several NO donors have been evaluated as a pharmacological alternative in the study of hypertension [30], our study is the first to show the anti-hypertensive effect of GSNO and DETA NONOate in the treatment of CKD-induced hypertension. 

Several other NO-donors, such as isosorbide dinitrate and nitroglycerine, rapidly release NO but can cause oxidative stress [31]. Therefore, to minimize these problems, we utilized innovative NO-donors GSNO and DETA NONOate. Indeed, our results revealed that both NO donors reduced but did not increase oxidative stress damage in the kidneys. Although GSNO is considered safe, it can still suffer from short half-lives and ineffective delivery [18,32]. Unlike the half-life of DETA, NONOate is up to 56 h [15] and GSNO decomposes in hours [33]. We, hence, generated GSNO-loaded nanoparticles to enhance the stability of GSNO and promote the conversion efficiency of GSNO into NO. Data obtained from this study indicated that GSNO-loaded nanoparticles have a similar anti-hypertensive effect compared to DETA NONOate at the same dosing interval, despite the latter showing a better renoprotection.

Although copper-involved nanoparticles have excellent physical and chemical properties [34], previous studies have shown that copper-based nanoparticles may induce oxidative stress damage to the liver, spleen, and kidney [35,36]. So far, no information exists on whether Cu/ZIF-8 nanoparticles can possess oxidase-like activity and cause the accumulation of reactive oxygen species (ROS) in tissues or organs. Of note is that this study only examined the kidney and found that GSNO-loaded Cu/ZIF-8 nanoparticles did not induce renal oxidative damage in control rats. However, there may be other important organs, such as liver and spleen, sensitive to Cu/ZIF-8 nanoparticle-related oxidative stress damage that remains to be determined in future studies.

At first sight, one might conclude that the BP-lowering effect of both NO donors in the young CKD rats is simply related to the vasodilator effects of NO released from these compounds. Interestingly, however, their beneficial effects are also associated to the reduction of oxidative stress and mediation of the RAS. In support of accumulating evidence that implicates oxidative stress in CKD and hypertension [11], adenine-treated CKD rats developed hypertension coinciding with enhanced oxidative stress, represented by 8-OHdG staining. Conversely, GSNO-loaded nanoparticle and DETA NONOate treatments are able to reduce BP and oxidative stress concurrently, suggesting their BP-lowering effects might be directly linked to oxidative stress.

Along with oxidative stress, the RAS plays a fundamental role in the development of hypertension and kidney disease [37]. Consistent with prior work showing the blocking of the classic RAS axis for the treatment of hypertension [37,38], GSNO-loaded nanoparticles protected against hypertension coinciding with the reduced expression of renin, ACE1, and AT1R in the kidneys. Similarly, DETA NONOate treatment downregulated renal AT1R expression. 

Moreover, GSNO-loaded nanoparticle and DETA NONOate treatments have differential effects on endogenous NO-generating systems; the plasma ADMA level was reduced by the former, while the latter caused a decrease in SDMA. Both ADMA and SDMA are well-known NOS inhibitors [29]. ADMA is extensively metabolized by DDAHs, whereas SDMA lacks appreciable metabolism and is almost completely eliminated by the kidneys. Considering DETA NONOate treatment restored the creatinine level back to normal, presumably the reduction of SDMA was due to the improvement of kidney function. Though GSNO-loaded nanoparticles had a neglectable effect on the protein abundance of DDAHs, a previous study reported that GSNO could increase DDAH activity to reduce ADMA [39]. It is possible that GSNO-loaded nanoparticles reduced ADMA via restoring DDAH activity inhibited by oxidative stress; however, this awaits further clarification. 

This study still has some limitations. In the view of diverse diversified biological activities of NO, the beneficial actions of NO donors on kidney injury and hypertension might be attributed to additional mechanisms involving other BP-controlling organs. In contrast to the constitutively expressed eNOS and nNOS, inducible NOS (iNOS) is undetectable under in the normal kidneys. We, hence, did not measure iNOS in the current study. However, iNOS can be induced under pathological conditions. Prior research showed that different NO donors affect transcriptional regulation of iNOS differently [40]. Our previous work indicated that iNOS inhibitor attenuated hypertension development in spontaneously hypertensive rats [41]; thus, further studies are required to evaluate whether anti-hypertensive effects of GSNO and DETA NONOate are related to the inhibition of iNOS. Another limitation is that we did not treat control rats with DETA NONOate as we used it as a positive control for comparison with GSNO-loaded nanoparticles. Nevertheless, it deserves further illumination as to whether the effect of DETA NONOate on control rats is similar to the hypotensive effect of GSNO-loaded nanoparticles on the controls. Moreover, we did not investigate additional dosing intervals of GSNO-loaded nanoparticles; whether nanoparticle-delivered GSNO can prolong retention time and produce long-term protection still awaits further investigation. Lastly, the results obtained from our study are promising for indicating that NO donors have beneficial actions on CKD-induced hypertension and kidney injury but are limited to testing in this model. More research is needed in other pediatric CKD models and in children before NO donors can be translated into a clinical reality. 

## 5. Conclusions

In summary, our work not only develops a nanotherapy for NO delivery, but also demonstrates that GSNO-loaded nanoparticles, same as DETA NONOate, protect against hypertension and kidney injury in a young rat model of CKD. Our data highlight the differential effects with two NO donors, GSNO and DETA NONOate, on endogenous NO-generating systems and the RAS components despite both having a similar BP-lowering effect. Our study has shown promising results, which lead us to develop novel NO delivery nanoparticles in an attempt to avert pediatric CKD and its complications in clinical practice.

## Figures and Tables

**Table 1:** Primary antibodies for Western blot.

Protein	Host	Company	Catalog No.	Dilution
eNOS	Mouse	BD Biosciences	BD610297	1:250
nNOS	Mouse	Santa Cruz	SC-5302	1:200
DDAH1	Mouse	Santa Cruz	SC-271337	1:500
DDAH2	Rabbit	Abcam	Ab184166	1:2000

eNOS = endothelial nitric oxide synthase; nNOS = neuronal nitric oxide synthase; DDA1 = dimethylarginine dimethylaminohydrolase-1; DDAH2 = dimethylarginine dimethylaminohydrolase-2.

**Table 2:** qPCR primer sequences.

Gene	5′ Primer	3′ Primer
Agt	5 gcccaggtcgcgatgat 3	5 tgtacaagatgctgagtgaggcaa 3
Renin	5 aacattaccagggcaactttcact 3	5 acccccttcatggtgatctg 3
PRR	5 gaggcagtgaccctcaacat 3	5 ccctcctcacacaacaaggt 3
ACE1	5 caccggcaaggtctgctt 3	5 cttggcatagtttcgtgaggaa 3
ACE2	5 acccttcttacatcagccctactg 3	5 tgtccaaaacctaccccacatat 3
AT1R	5 gctgggcaacgagtttgtct 3	5 cagtccttcagctggatcttca 3
MAS	5 catctctcctctcggctttgtg 3	5 cctcatccggaagcaaagg 3
R18S	5 gccgcggtaattccagctcca 3	5 cccgcccgctcccaagatc 3

## Data Availability

Data are contained within the article.
